# Amorphous selenium nanoparticles: synthesis strategies and multifunctional applications

**DOI:** 10.1039/d6ra04432h

**Published:** 2026-07-21

**Authors:** Pratibha Chahal, Avinash Singh

**Affiliations:** a Department of Chemistry, SRM University Delhi-NCR Sonepat Haryana 131029 India singhavi06@gmail.com

## Abstract

Selenium is a vital trace element involved in many biological processes. In recent years, amorphous selenium nanoparticles (a-SeNPs) have gained considerable interest for their better biocompatibility, reduced toxicity, and better functionality than the crystalline form. Because of these properties, a-SeNPs are being increasingly investigated for various applications. a-SeNPs can be prepared by chemical, hydrothermal, microwave-assisted, and biological synthesis, with the latter providing better stability and biocompatibility. Their size, shape, and amorphous nature have a profound impact on their physico-chemical and biological properties. This review discusses different synthesis strategies and highlights the multifunctional applications of a-SeNPs, including antioxidant, antibacterial, anticancer, bioimaging, biosensing, agricultural, and photocatalytic applications along with future perspectives in this field.

## Abstract 2

Synthesis strategies and applications of a-Se nanoparticles.

## Graphical abstract

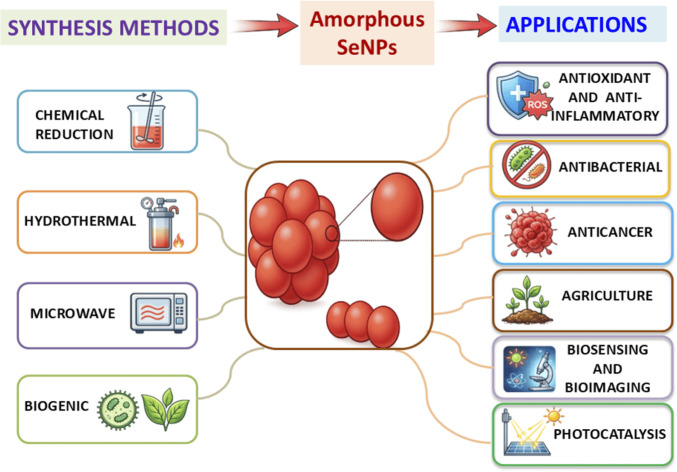

## 1 Introduction

**Fig. 1:**
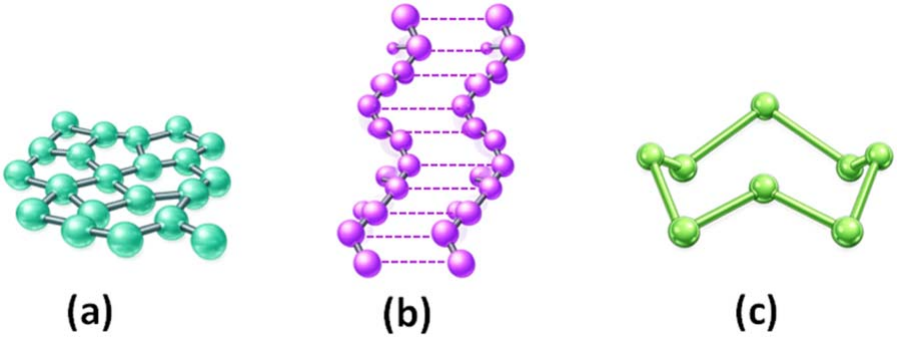
Crystal structural representation of Se (a) a-Se, (b) t-Se, and (c) m-Se.

Scientists have been researching selenium (Se) over the past 200 years due to its numerous useful properties and its indispensable roles in living systems. Se was identified by Jons Jakob Berzelius in 1817, and its name was derived from the Greek word Selene, meaning “moon”. Although this element was originally considered primarily toxic, selenium was eventually identified as an important micronutrient in the middle of the twentieth century. Plants are the major source of food, as they absorb inorganic Se from the soil and convert it to selenomethionine (SeMet), which is then incorporated into the food chain.^1^ Se is a semi-metallic element similar to sulfur and tellurium in chemical properties and appears as a red powder, black vitreous and metallic grey crystalline solid.^2^ It can exist in oxidation states of −2, 0, +4 and +6 and forms common inorganic species such as selenide, selenite and selenate.^3,4^ Se also occurs in three allotropes: monoclinic (m-Se), trigonal (t-Se) and amorphous (a-Se) (Fig. 1). The most stable crystalline form of selenium is t-Se, and it consists of parallel helix chains of Se atoms. The atoms are held together along the chains by strong covalent bonds, but the van der Waals interactions between the neighbouring chains are weak. The m-Se exists in three forms (α, β, and γ), each consisting of eight-membered Se_8_ rings similar to those found in sulfur.^2,5^ a-Se, on the other hand, has a disordered structure and has a relatively low glass transition temperature (∼31 °C), making it metastable.^6^ This type of structural disorder offers a-Se a high surface energy, tunable electronic behaviour, and high photoconductivity, which are useful in xerography, photovoltaic devices, and medical imaging detectors. More importantly, the lack of long-range atomic order leads to a high density of structural defects such as dangling bonds, undercoordinated selenium atoms, vacancies, and strained Se–Se bonds.^7^ All these defect-rich surfaces provide many chemically active sites, which can promote adsorption and redox reactions at the interfaces. The disordered atomic network can also localize charge carriers, promote electron transfer to molecular oxygen to generate reactive oxygen species (ROS), and increase the interactions with biomolecules (proteins, lipids, nucleic acids, and cellular membranes).^8,9^ In addition, a-Se is metastable and may undergo structural rearrangement or partial oxidation/dissolution under biological conditions, releasing reactive selenium species and contributing further to redox activity. Together, these physicochemical properties provide the basic mechanism by which a-Se often exhibits better antioxidant, antibacterial, anticancer, and photocatalytic activities than c-Se.

In biological systems, Se performs essential functions by being incorporated into selenoproteins containing selenocysteine or SeMet. More than 25 selenoproteins, such as thioredoxin reductases, selenoprotein P, and glutathione peroxidises have been identified and are involved in maintaining cellular redox balance, antioxidant protection, and immune homeostasis. Se compounds can also influence gene expression, DNA repair, and oxidative stress pathways, although their beneficial and toxic effects depend strongly on their chemical form and dosage. Due to the narrow therapeutic index of most selenium compounds, much focus has been placed on safer selenium-based systems.

Recent developments in nanotechnology have sparked growing interest in SeNPs as safer, more efficient selenium platforms. SeNPs exhibit better biocompatibility, lower toxicity, and enhanced antioxidant and anticancer properties compared to traditional selenium compounds. Their small size allows them to be readily absorbed by the cell and enhances their interactions with biological systems. Plant extracts or microbial subcomponents have attracted much interest in green synthesis approaches due to their properties as reducing and stabilising agents, allowing the production of eco-friendly SeNPs in the absence of toxic reagents. a-SeNPs have attracted particular interest in these Se nanostructures, as the intrinsic physicochemical properties of a-Se are further enhanced by nanoscale engineering, leading to increased bioavailability and improved interactions with biological systems. Such nanomaterials can also slowly rearrange into more crystalline structures because a-Se is metastable;^10^ therefore, the rate of this structural transition can vary with factors such as temperature, precursor concentration, and reaction environment. Surface capping with proteins, polysaccharides, glutathione (GSH) or polymeric substances is thus frequently utilised to control the amorphous structure.^89^ Fine control of particle size, morphology, and crystallinity is provided by advanced synthesis techniques, including hydrothermal processes, sonochemical methods, photochemical reduction, electrochemical methods, pulsed-laser methods, and vapour-phase methods.^2^ These improvements enable the production of stable, uniform a-SeNPs for biomedical and technological applications. The purpose of this review is to combine the chemical properties, biological functions, and nanoscale behaviour of a-Se to emphasise its increasing importance in biomedical science. The antioxidant, antibacterial, anticancer, bioimaging, biosensing, agriculture and photocatalytic applications of a-SeNPs are given special focus to provide a concise and broad overview to scientists looking to find out more about the growing possibilities of Se nanotechnology.

## 2 Synthesis of selenium nanoparticles

SeNPs are synthesised *via* biological or chemical methods using different selenium precursors such as sodium selenite (Na_2_SeO_3_), sodium selenate (Na_2_SeO_4_), selenium dioxide (SeO_2_), selenious acid (H_2_SeO_3_). Biological approaches use microorganisms, plant extracts, or biomolecules as natural reducing and stabilising agents, whereas chemical methods rely on synthetic reducing agents and capping agents for SeNPs formation. The synthesis routes are further distinguished based on energy source or experimental setup, such as hydrothermal or microwave-assisted synthesis, distinguishes these methods. This classification organises helps to organise the different synthesis pathways discussed in the following sections.

### 2.1 Chemical reduction

Out of the various preparation strategies reported, selenium salt reduction is one of the most commonly used and easiest methods for synthesising SeNPs. In this procedure, the precursors of selenium are reduced to elemental selenium with either chemical reducing agents, such as ascorbic acid,^11^ or naturally occurring substances in plants and microorganisms.^12^ It has been noted that SeNPs prepared with natural compounds tend to be less toxic than those prepared by purely chemical methods. In traditional chemical reduction methods, different stabilising and capping reagents are used to control particle growth and ensure the formation of stable colloidal SeNPs. They are synthetic polymers, such as polyvinylpyrrolidone (PVP)^13^ and polyethylene glycol (PEG),^14^ proteins such as bovine serum albumin (BSA),^15^ and small biomolecules such as folic acid.^16^ Moreover, polysaccharides have also been found to work as reducing and stabilising agents, as demonstrated by Liu *et al.*,^17^ thereby increasing the stability of SeNPs for biomedical use. In another study, Zhang *et al.*^18^ prepared polysaccharide-stabilized SeNPs using *spirulina* polysaccharide, Na_2_SeO_3_, and ascorbic acid. The resulting SeNPs have a spherical from, an average size of 73 nm and had good storage stability, lasting up to 75 days at 4 °C. Similarly, Boroumand *et al.*^19^ prepared spherical SeNPs through reduction of Na_2_SeO_3_ with ascorbic acid and reported an average particle size of about 66 nm. These NPs produced a moderate cytotoxic effect on fibroblast cells, demonstrating their biological activity. El-Ghazaly *et al.*^20^ synthesised SeNPs with SeO_2_, KBH_4_, PVP under ice-cold conditions. The formation of NPs was indicated by a characteristic orange hue, suggesting the creation of a-Se. The resulting a-SeNPs were spherical with sizes around 10–13 nm, as confirmed by TEM analysis.

#### 2.1.1 Microwave-assisted method

**Fig. 2:**
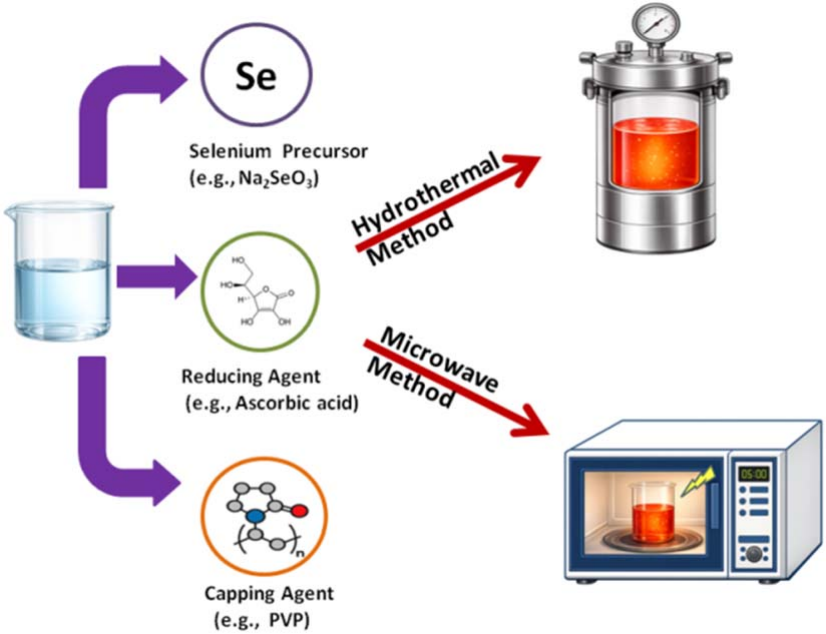
Synthesis of SeNPs using hydrothermal and microwave methods.

Microwave-assisted synthesis is a viable alternative to typical heating techniques for preparing SeNPs. Under this technique, reaction systems containing selenium precursors and reducing agents are reproducibly heated with microwave radiation, resulting in rapid, homogeneous heating of the media (Fig. 2). Consequently, the reactions are fast and can be completed within a relatively short time and with relatively low energy.^21^ This technique is considered an environmentally friendly synthesis route due to its operational simplicity, low processing time, and high product yield.^22^ The nature of synthesised SeNPs depends on several factors, including irradiation time, microwave power, solution pH, precursor concentration, and the type of reducing or stabilising agents used. Mellinas *et al.*^23^ reported that *Theobroma cacao* L. bean shell extract (CBS) was utilized as a reducing and stabilising agent in the microwave-assisted formation of SeNPs. The parameters maximised in a central composite design included irradiation time, microwave power, and precursor amount. Optimisation of conditions led to the production of SeNPs of approximately 42 nm using 15.6 min irradiation time, 788.6 W power, 0.14 g Na_2_SeO_3_, and 50 mL extract, which were uniformly distributed as spherical particles ranging from 1 to 3 nm, with stability lasting up to 2 months. Likewise, Fardsadegh *et al.*^24^ established a microwave-assisted synthesis process employing *Pelargonium zonale* leaf extract, utilising response surface technique to optimise the process. Optimisation of the system was achieved after 4 min of irradiation, yielding NPs with an average size of about 50 nm and a zeta potential of −24.6 mV, indicating high colloidal stability. In a different study, SeNPs were produced through the application of microwave irradiation, where the source of selenium was SeO_2_, the reducing agent was hydrazine, and the stabilising agent was SDS and irradiation with approximately 750 W over a short period produced dark-coloured SeNPs with fairly homogenous distribution of size.^25^ Moreover, the synthesis of SeNPs using microwave-assisted using glucose as a green reducing agent has also been reported, thus allowing rapid development of less toxic SeNPs.^26^

#### 2.1.2 Hydrothermal method

The hydrothermal technique offers significant versatility for producing biologically compatible SeNPs across a wide size range.^27^ Though relatively underutilised, literature demonstrates its efficacy in generating SeNPs as small as 10–20 nm through reactions conducted in a sealed autoclave under controlled temperature and pressure (Fig. 2). This process enables precise control over nucleation, growth, and the resulting NP structure. Most commonly, hydrothermal reactions yield SeNPs in the a-Se form, with temperature, precursor concentration, and reaction duration determining their structural characteristics.^28^ For example, Zeng *et al.*^29^ prepared SeNPs by dissolving selenium in ethylenediamine, autoclaving at 160 °C for 2 h, and cooling to ambient temperature, yielding a homogeneous brown solution. The product was further cooled to −18 °C to form a-SeNPs. Similarly, Chen *et al.*^30^ obtained spherical a-SeNPs (∼280 nm) confirmed as amorphous *via* diffuse rings in electron diffraction. Other studies show the ability to tune particle size, charge, and dispersity. Hatami *et al.*^31^ reported SeNPs with an average size of 110 nm, a zeta potential of 44.9 mV, and a polydispersity index of 0.226. Abbasian *et al.*^32^ achieved synthesis using coffee bean extract under moderate conditions in just 15 min. Reduction of Na_2_SeO_3_ with hydrazine chloride can yield even smaller (∼15 nm) SeNPs, highlighting the methods capacity for nanoscale production. Hydrothermal synthesis also allows flexibility in choosing reducing agents and reaction systems. Morphology and phase control are possible with compounds such as glucose or l-cysteine, enabling the fabrication of structures such as nanorods and t-Se phases.^33^ Often, an early amorphous stage transitions to more stable crystalline forms,^34^ which is significant because selenium's optical and photoconductive properties are strongly dependent on phase and structure.^35^

### 2.2 Biogenic reduction

Biogenic synthesis has become one of the environmentally friendly and sustainable methods of preparing SeNPs. The biological systems in this approach use plant extracts or microorganisms to reduce selenium ions to NPs under mild reaction conditions, usually in aqueous media.^36^ The poor cellular uptake of inorganic selenium forms is a key issue with their use, limiting their potential for biological applications. Biologically synthesised SeNPs, however, are more stable and more compatible with living systems.^37^ Usually, chemical pathways require harsh and toxic chemicals that limit their biomedical applications, whereas plant-mediated synthesis offers a safer alternative.^38^ Plant-based synthesis can be used to address this issue, since plant extracts are rich in natural components like as phenolic compounds, proteins, flavonoids, enzymes, and polysaccharides that serve as reducing and stabilising agents and help prevent clumping of NPs.^39^ Mechanistically, these phytochemicals donate electrons to reduce selenium ions (Se^4+^) to elemental selenium (Se^0^) for nucleation. The low-selenium atoms are then pooled to form nanoclusters, and further growth is regulated to produce stable NPs. In the process, hydroxyl and carbonyl groups (*e.g.* flavonoid-like quercetin or catechin) can act as functional groups to reduce Se *via* electron transfer and can be oxidised to quinone derivatives. At the same time, these biomolecules bind onto the NPs surface and serve as stabilising layers, stabilising the particles, preventing agglomeration, and guiding morphology. The reaction conditions, such as pH, also affect the particle size and shape by modulating the reduction efficiency and crystallisation behaviour.

Plant-mediated synthesis is more feasible than microbial methods, as it does not require culture maintenance and is less complex.^40^ It is cost-effective and easy, does not require controlled conditions, generates less waste, uses fewer chemicals, and consumes fewer chemicals.^41^ Other biological sources that have been investigated include fungi, proteins, algae, and bacteria, but plant extracts are the most popular because they are readily available and environmentally friendly. A variety of parts of plants including *Terminalia arjuna*,^42^*Allium sativum*,^43^*Castanea dentata*,^40^*Emblica officinalis*,^44^*Moringa oleifera*,^45^*Cassia auriculata*^46^ and *Undaria pinnatifida*,^47^ have been reported on this purpose. For example, it has been reported that plant-mediated synthesis using *Diospyros montana* bark extract has been shown, in which phytochemicals can reduce selenious acid under mild conditions to form.^48^ Likewise, leaf extract of *Nyctanthes arbor-tristis* has also been used to synthesise stable, spherical SeNPs, with the plant phytochemicals serving as reducing and stabilising ions, resulting in a stable, spherical shape with high yield of SeNPs.^49^

## 3 Characterization of selenium nanoparticles

**Fig. 3:**
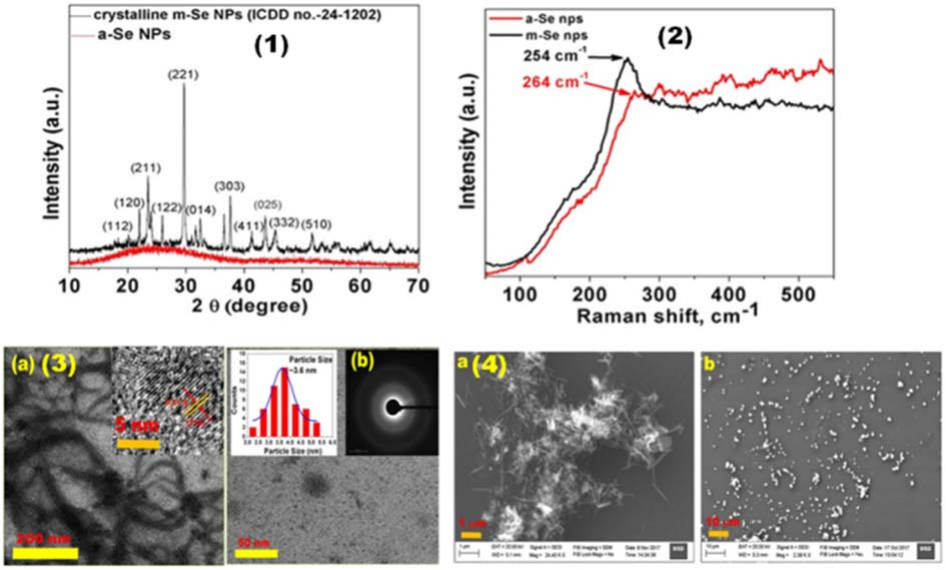
(1) XRD pattern of c-SeNPs and a-SeNPs, (2) Raman spectra of both SeNPs, (3a) TEM image of c-SeNPs, (3b) TEM and SAED image of a-SeNPs, (4a) SEM image of c-SeNPs, (4b) SEM image of a-SeNPs. This figure has been reproduced from ref. 4 with permission from IOP, copyright 2018.

Following synthesis, reliable structural characterization is crucial to confirm whether SeNPs exist in the amorphous or crystalline phase, as their crystallinity strongly influences physicochemical and biological activity. As no single characterization technique can unambiguously differentiate amorphous and crystalline SeNPs, a combination of X-ray diffraction (XRD), Raman spectroscopy, SEM/HRTEM, and selected area electron diffraction (SAED) is typically employed to provide complementary information on their crystallinity, atomic ordering, and morphology. Singh *et al.*^4^ demonstrated that starch effectively inhibited the crystallization of SeNPs, yielding predominantly a-SeNPs (Fig. 3).

XRD analysis showed a broad peak at ∼25° (2*θ*) instead of the characteristic crystalline reflections, while Raman spectra confirmed the amorphous phase through broadened vibrational features compared to the 254 and 264 cm^−1^ bands of crystalline selenium. HRTEM further revealed lattice fringes with an interplanar spacing of 4.31 Å for c-SeNPs, whereas a-SeNPs (∼3.6 nm) exhibited diffuse SAED rings and globular “cotton-plug” morphology in SEM. Further advancing the controlled synthesis of SeNPs, Guleria *et al.*^6^ demonstrated that room-temperature ionic liquids (RTILs) enabled precise control over nanoparticle phase and morphology by varying the reaction temperature. SeNPs synthesised at 35 °C exhibited a broad amorphous XRD profile and a characteristic Raman band at ∼256 cm^−1^, while NPs prepared at 80 and 120 °C displayed sharp trigonal XRD reflections together with a Raman band at ∼234 cm^−1^. The intermediate SeT50 sample exhibited both 234 and 256 cm^−1^ bands, indicating the coexistence of amorphous and crystalline phases. TEM, FESEM and SAED analyses consistently revealed spherical NPs with diffuse diffraction rings for the amorphous phase and rod- or needle-like nanostructures with sharp diffraction rings for the crystalline phase. Raman mapping further confirmed the predominance of the 256 cm^−1^ band in freshly synthesised SeT35 NPs and demonstrated that the RTIL remarkably delayed the amorphous-to-crystalline transformation for more than one month. More recently, Ferroudj *et al.*^50^ directly compared red (amorphous) and grey (crystalline) SeNPs, reporting a broad diffuse XRD halo between 20–30° (2*θ*) and weak, broadened Raman bands in the 250–255 cm^−1^ region for red SeNPs, whereas grey SeNPs exhibited sharp XRD reflections corresponding to trigonal selenium, along with an intense Raman peak at 230–235 cm^−1^ assigned to the A_1_ stretching mode of ordered Se–Se chains. SEM observations further revealed predominantly spherical morphology for a-SeNPs and elongated needle-like structures for c-SeNPs, highlighting the close relationship between crystallinity and NPs morphology.

## 4 Nanotoxicity of selenium nanoparticles

The nanotoxicity and biocompatibility of a-SeNPs are important considerations for evaluating their potential biomedical applications. Compared with inorganic selenium compounds such as sodium selenite, SeNPs have been shown to be less toxic and more biocompatible.^4^ Several factors, such as dose, structural phase, particle size, surface functionalization, route of administration and duration of exposure, however, have a significant impact on their biological effects. The structural phase of SeNPs is particularly significant, as it can directly influence their biological activity and safety profile. The a-SeNPs (SeT35) synthesised at 35 °C demonstrated the highest selective cytotoxicity against A549 lung cancer cells after 48 h with an exposure concentration of 100 µg mL^−1^, with negligible toxicity towards normal WI26VA4 lung epithelial cells. The SeT50 NPs with both amorphous and crystalline phases exhibited selective anticancer activity, while the predominantly crystalline SeT80 and SeT120 NPs exhibited little or no cytotoxicity under identical conditions. This study suggests that the amorphous phase is more effective for anticancer activity than the crystalline phase, while retaining good biocompatibility with normal cells.^6^ Further evaluation of the cytotoxicity of RTIL-stabilized a-SeNPs at 25, 50, and 100 µg mL^−1^ against A549 lung cancer cells showed increased cytotoxicity with increasing concentration, whereas INT407 normal epithelial cells showed minimal changes in viability across the concentration range. However, selenium precursor (SeO_2_) showed toxicity to both normal and cancer cells, the highest being anticancer effect of a-SeNPs at 100 µg mL^−1^. When applied to normal L929 fibroblast cells under study, SeNPs caused a dose- and time-dependent reduction in cell viability, with chitosan-coated SeNPs being significantly more toxic (cell viability of 170 ppm after 72 h was 67.4%), while exposure to 170 ppm for 24 h caused only a slight reduction in cell viability.^19^*In vivo*, the SeNPs were shown to be non-toxic and therapeutically effective up to 3–4 mg Se kg^−1^, but were toxic at 5–6 mg Se kg^−1^ and were associated with decreased survival in healthy mice. These indicate that the safe dose range is approximately 3–4 mg Se kg^−1^ with indications of toxicity beginning to occur at 5–6 mg Se kg^−1^ and beyond.^51^

## 5 Applications of selenium nanoparticles

The applications of SeNPs have been extensively explored because of their distinctive physicochemical behaviour and biological activity, and they can be broadly classified into biomedical, environmental, and agricultural applications. In biomedical applications, SeNPs exhibit antioxidant, anticancer, antibacterial, bioimaging, and biosensing properties, owing to their high biocompatibility, low toxicity, and strong interactions with biological molecules. Environmental applications mainly involve photocatalytic degradation of organic pollutants through light-induced generation of ROS. In the agricultural sector, SeNPs serve as controlled-release sources of selenium, improving plant growth, stress tolerance, and nutrient uptake at low concentrations. This classification provides a systematic understanding of the multifunctional nature of SeNPs, which is discussed in detail in the following sections.

### 5.1 Antioxidant

The production of ROS, which includes superoxide anion (O_2_^−^), hydrogen peroxide (H_2_O_2_), hydroxyl radicals (OH˙), and singlet oxygen (^1^O_2_), and the body's antioxidant defence system, is needed in balance to ensure normal cellular functioning. The imbalance in this condition leads to oxidative stress, which damages biomolecules such as lipids, proteins, and DNA, thereby contributing to diverse disorders, including neurodegenerative diseases, inflammation, heart-related issues, and cancer.^52^ These reactive species cause cellular damage, and antioxidant compounds help cells survive by neutralising them and maintaining redox balance. SeNPs have demonstrated good antioxidant properties among other nanomaterials. According to Qamar *et al.*,^53^ a-SeNPs exhibit dose-dependent antioxidant activity in multiple assays, including FRAP, DPPH, hydrogen peroxide (H_2_O_2_), superoxide (O_2_^−^), and nitric oxide (NO) scavenging. Biogenic amorphous TA-SeNPs (17.6 nm) showed superior antioxidant performance, especially at higher concentrations (40–100 µg mL^−1^), achieving more than 90% scavenging in DPPH and NO assays and, in some cases, outperforming ascorbic acid. In contrast, c-SeNPs (109 nm) showed slightly better activity only at lower concentrations (2.5–5 µg mL^−1^). FRAP results indicated that c-SeNPs had higher reducing power at low doses, while TA-SeNPs became more effective at higher concentrations. In H_2_O_2_ and superoxide scavenging assays, both NPs showed concentration-dependent activity. Cellular studies further revealed that TA-SeNPs effectively reduced intracellular ROS at low doses (5–10 µg mL^−1^), whereas c-SeNPs required higher concentrations (25–100 µg mL^−1^) to achieve similar effects. Overall, the smaller size, amorphous nature, and phytochemical capping make TA-SeNPs more effective antioxidants compared to crystalline SeNPs. Similarly, Nagaraj *et al.*^54^ reported that plant-mediated a-SeNPs exhibited strong antioxidant activity in DPPH and ABTS assays, showing concentration-dependent radical scavenging with EC_50_ values of 68.5 µg mL^−1^ and 73.3 µg mL^−1^, respectively. The enhanced antioxidant effect was attributed to the combined action of selenium and surface-bound phytochemicals such as rutin, gallic acid, and quercetin, which act as electron donors and stabilize the NPs. Furthermore, biological studies demonstrated that these NPs effectively reduced oxidative stress by lowering ROS levels and restoring antioxidant enzyme activities (SOD, CAT, and GPx), confirming their strong *in vitro* and *in vivo* antioxidant potential. In a related study, Senthamaraikannan *et al.*^55^ demonstrated that a-SeNPs (CNH-SeNPs) also possess strong antioxidant activity, showing 80.72%, 84.67%, and 74.28% scavenging in DPPH, ABTS, and H_2_O_2_ assays, respectively, at 50 µg mL^−1^. Upon PEG coating, the resulting nanocomposite (CNH-SeNC), which exhibited increased crystallinity, showed slightly improved antioxidant activity with values of 87.08%, 86.24%, and 81.44% in the respective assays, indicating that surface modification enhances antioxidant performance. Overall, these studies show that amorphous and plant-mediated SeNPs generally exhibit superior antioxidant activity and that surface functionalization further improves their activity and stability. Likewise, Guleria *et al.* demonstrated that a-SeNPs, with the aid of RTIL exhibited excellent antioxidant activity in ABTS assay with an IC_50_ value of 0.55 mg mL^−1^ and 65% scavenging capacity at 0.75 mg mL^−1^. It was suggested that the RTIL stabilizes the amorphous phase for long periods of time (>1 month) to maintain the antioxidant activity of the NPs.^56^ Amir Ali *et al.*^57^ reported that phytogenic SeNPs combined with light-treated *Caralluma tuberculata* callus extract showed strong antioxidant activity. At 800 µg mL^−1^, the extract exhibited a reducing power of about 26.29% and ABTS radical scavenging of 42.51%. It also showed effective H_2_O_2_ scavenging (37.26%) and OH˙ scavenging (40.23%). The total antioxidant capacity reached 71.66% in the phosphomolybdate assay, which was close to that of ascorbic acid. This improvement was linked to higher levels of polyphenols and flavonoids induced by SeNPs and light stress. Abadi *et al.*^58^ reported that chitosan-stabilized SeNPs exhibit moderate antioxidant activity, which increases after quercetin loading. In the DPPH assay, quercetin-loaded SeNPs reached nearly 80% radical scavenging at 320 µg mL^−1^, while unloaded SeNPs showed about 50% activity, and free quercetin remained high. Reducing power results showed improvement only at higher concentrations, indicating that the antioxidant effect depends mainly on sufficient quercetin release and NPs dose.

### 5.2 Anticancer

**Fig. 4:**
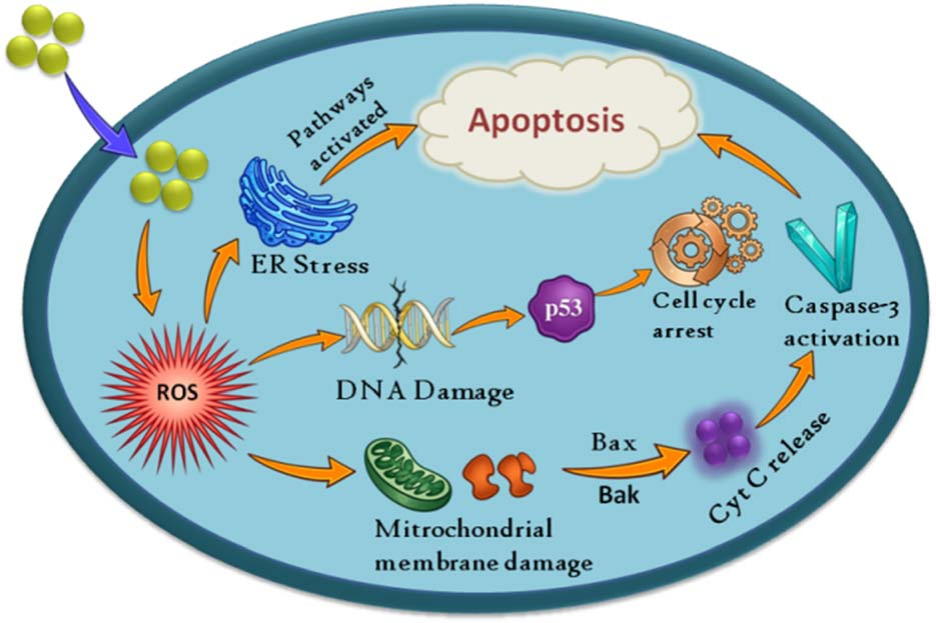
Schematic representation of the anticancer mechanism of SeNPs.

Over the past few years, SeNPs have gained popularity in anticancer therapy for breast, colorectal, lung, liver, cervical, and other cancers, driven by extensive *in vitro* and *in vivo* research.^2^ Traditional selenium preparations, such as elemental selenium and organic selenium compounds, are usually dose-dependent and poorly absorbed, limiting their therapeutic application. Conversely, nano-selenium exhibits better dispersion, controlled reactivity and cellular uptake, selective toxicity toward cancer cells, and reduced harm to normal cells. A number of reports have demonstrated that SeNPs surface-engineered induce cancer cell death primarily *via* oxidative stress, mitochondrial dysfunction, DNA damage, and apoptosis as shown in Fig. 4. Additional functionalization with natural compounds, polysaccharides, peptides, or targeting ligands enhances stability, reduces aggregation, and improves anticancer efficacy.

Guleria *et al.*^59^ found that predominantly a-SeNPs synthesised in room-temperature ionic liquids (RTILs), such as [EOHMIM][BF_4_], [EMIM][BF_4_], and [EMIM][MS], exhibited dose-dependent anticancer effects against A549 cells at 25, 50, and 100 µg mL^−1^. The most active NPs were those prepared in [EOHMIM][BF_4_] with an average size of 32 nm, followed by [EMIM][BF_4_] (∼57 nm) and [EMIM][MS] (∼60 nm), showing a clear size-dependent behaviour. The selective toxicity was confirmed, as cancer cells shrank and detached whereas normal cells remained unaffected. Stability studies further revealed that SeNPs prepared in [EMIM][MS] showed minimal size variation over time compared to the other RTIL systems. The research also indicated that these NPs were initially amorphous but gradually crystallized over time, becoming more stable and less reactive. Overall, the results suggest that smaller a-SeNPs exhibit better anticancer performance, and stabilization of the amorphous phase is important to retain their biological activity. Similarly, SeNPs synthesised at 35 °C and 70 °C were mainly amorphous, while SeNPs synthesised at 120 °C were mainly trigonal crystalline with minor monoclinic phase. The predominantly a-SeNPs showed significantly higher anticancer activity, with ∼80% (SeT35) and ∼40% (SeT70) cell death in A549 cells compared to the predominantly crystalline SeNPs (SeT120) which caused only ∼10–15% cell death. Furthermore, the a-SeNPs had virtually no toxicity to normal intestinal epithelial cells at 100 µg mL^−1^, emphasizing the need to maintain the amorphous form for effective and selective anticancer activity.^60^ Al-Duais *et al.*^61^ reported the anticancer potential of a-SeNPs synthesised by reducing Na_2_SeO_3_ using curcumin and stabilising them with the biopolymer fucoidan. These NPs exhibited high cytotoxicity against colorectal cancer cells, with IC_50_ values of 27.16 mg L^−1^ and 78.28 mg L^−1^ in CaCo-2 and HT-29 cells, respectively. Following fucoidan coating, the IC_50_ values were 10.35 mg L^−1^ and 19.44 mg L^−1^, which outperformed cisplatin. The NPs inflicted serious DNA damage, as the comet assay revealed almost 43% tail DNA and induced apparent apoptotic and autophagic alterations. They found that their anticancer activity was primarily linked to increased cellular uptake, ROS generation, and mitochondrial damage, with limited toxicity to normal cells. Alsubaie *et al.*^62^ also compared the cytotoxicity of quercetin-loaded SeNPs after 48 h of treatment; these NPs showed dose-dependent cytotoxicity with an IC_50_ of about 51 µg mL^−1^, compared to cisplatin, which had an IC_50_ of 18 µg mL^−1^. There was distinct apoptotic morphology and a marked rise in the sub-G0/G1 cell population in treated cells. The NPs caused prolonged intracellular ROS production, reaching levels almost 3 times higher at 48 h, leading to severe DNA damage, evidenced by longer comet tail lengths of approximately 15.7 µm compared to 12.3 µm with cisplatin. Evidence of ROS-mediated mitochondrial apoptosis was demonstrated by upregulation of Bax and cleaved caspase-3, and by inhibition of Nrf-2. a-SeNPs have also been investigated as gene-based cancer therapy. a-SeNPs are functionalized with peptides (LP-SeNPs) and were developed as safe gene carriers for triple-negative breast cancer. These NPs had sizes of 48–68 nm, high stability, and a high siRNA binding efficiency. Loaded with Src-siRNA, they demonstrated 70% Src protein knockdown in MDA-MB-231 cells and were comparable to lipofectamine, with significantly lower toxicity. Cellular uptake was 24%, NPs were non-toxic up to 8 µM, and uptake was primarily *via* energy-dependent macropinocytosis. The targeted delivery also increased anticancer activity.^63^ Se@MONs@LXL-1 aptamer-functionalized NPs, 80–95 nm in diameter, were found to have a 5.32-fold greater cellular uptake in MDA-MB-231 cells than non-targeted systems. The organosilica shell was destroyed, and the SeNPs were released under tumour-like conditions (pH 6.0 and 10 mM GSH), resulting in mitochondrial damage due to ROS. The targeted system demonstrated better anticancer activity with an IC_50_ of 71.13 + 3.22 µg mL^−1^, relative to 161.20 + 8.15 µg mL^−1^ of non-targeted NPs and low levels of toxicity to normal cells.^64^

### 5.3 Antibacterial

Microbe infectivity has continued to pose a significant challenge to human health, and recently, the widespread use of antibiotics has increased the rate of emergence of antimicrobial resistance (AMR).^65^ As a result, many bacterial and fungal infections that were previously easy to treat have become increasingly difficult to manage.^66,67^ Traditional drug development strategies, mainly based on modifying existing antibiotics, are struggling to keep pace with rapidly evolving resistance mechanisms.^68^ Consequently, alternative antimicrobial approaches are urgently needed. Nanomaterials are increasingly being explored for antimicrobial applications because of their distinctive chemical and physical properties and ability to act through multiple antimicrobial mechanisms. Among them, SeNPs have attracted considerable attention owing to their effective antimicrobial potential and relatively low toxicity compared with other nanomaterials, such as silver NPs.^69–72^ Studies have shown that SeNPs can inhibit the growth of both Gram-positive and Gram-negative bacteria, primarily by damaging membranes, generating ROS, and disrupting essential cellular processes.^73–77^ Sharmila *et al.*^78^ synthesised a-SeNPs using the medicinal plant *Aporosa cardiosperma* and evaluated their antibacterial activity against several pathogenic bacteria. They reported strong antibacterial effects, with the highest inhibition observed against *Klebsiella pneumoniae*. At a concentration of 100 µg mL^−1^, a zone of inhibition of 22.67 ± 0.33 mm was recorded. They suggested that this antibacterial behaviour may result from interactions between the NPs and bacterial cell membranes, which can disturb essential cellular processes and inhibit microbial growth. Similarly, Ruiz-Fresneda *et al.*^79^ investigated the antibacterial activity of a-SeNPs (50–90 nm) with different surface coatings against *Stenotrophomonas bentonitica* and *Lysinibacillus sphaericus*. Among the tested materials, SeNPs coated with undefined showed the strongest antibacterial effect, causing approximately 85–91% bacterial cell death at 100 µM. The treatment also resulted in a significant increase in ROS production (up to ∼299% compared with untreated cells) and notable DNA degradation (around 80%). These data suggest that a-SeNPs have antibacterial effects using multiple mechanisms, including oxidative stress, membrane disruption and intracellular damage, ultimately reducing bacterial viability. Aneesha *et al.*^80^ used *Brassica oleracea* (broccoli) extract for the green synthesis of a-SeNPs, which showed good colloidal stability. The synthesised NPs exhibited strong antimicrobial activity against *Escherichia coli*, *Aspergillus niger*, and *Staphylococcus aureus*, with a maximum inhibition zone of 23.67 ± 0.58 mm, along with notable antioxidant and antibiofilm activity.

### 5.4 Bioimaging

Medical imaging plays a critical role in the study of disease within the body, and most modern imaging techniques suffer from low contrast, poor sensitivity, and side effects from the contrast agent. Various nanomaterials have been tried to enhance the quality of imaging over the years.^81^ Although gold, silver, and other metal NPs exhibit strong optical properties, concerns about toxicity, signal instability, and low biodegradability have limited their long-term applications.^82–84^ a-SeNPs are much more biocompatible and generate stable optical signals in biological systems.^85^ The ability to emit light without the need for additional fluorescent dyes or radioactive labels is an important advantage, as it enables SeNPs to be used for imaging. This minimises the background noise and prevents extra toxicity. It has been demonstrated that SeNPs can easily be observed in living cells during cell growth and division. Further uses of these NPs have demonstrated potential in cancer imaging and biosensing. They demonstrate high selectivity for sensing cancer biomarkers at very low concentrations and are tuned to optical responses, as reported in the study.^86^

According to Khalid *et al.*,^87^ a-SeNPs exhibit broad photoluminescence spanning 400–700 nm, with a sharp emission peak at 580 nm. Fluorescence of the NPs was seen to be stable with an intensity of between 10–30 counts per second and an average lifetime of approximately 4.5 ns, which is a sign of good photostability. They were effectively imaged within fibroblast cells without exogenous labelling, and over 70% cell viability was maintained, validating their low toxicity and biocompatibility for imaging and biosensing studies. In a separate study, Liu *et al.*^88^ synthesised a-SeNPs as dark-field imaging light-scattering probes. SeNPs exhibited a unique green scattering spectrum with a maximum at 570 nm, and thus the NPs could be easily seen at the single-particle level. The signal from a single SeNPs was quite strong and comparable to approximately 1.34 × 10^−5^ molecules of fluorescein. The NPs remained in solution for several days and did not exhibit any obvious toxicity. Once they had been functionalized with aptamers, they could specifically target nucleolin-overexpressing HEp-2 cancer cells and produced strong, localised signals on the cell surface, indicating that they could be used for cancer cell imaging.

GSH-capped SeNPs were used by Korany *et al.*^89^ for tumour imaging, radiolabeled with technetium-99m. The NPs produced had an amorphous core with a median size of approximately 21 ± 5 nm, a high radiochemical stability of approximately 97%, and good *in vivo* biodistribution. They could accumulate in tumour tissues and showed good imaging contrast, demonstrating their potential for cancer diagnostics.

### 5.5 Biosensing

**Fig. 5:**
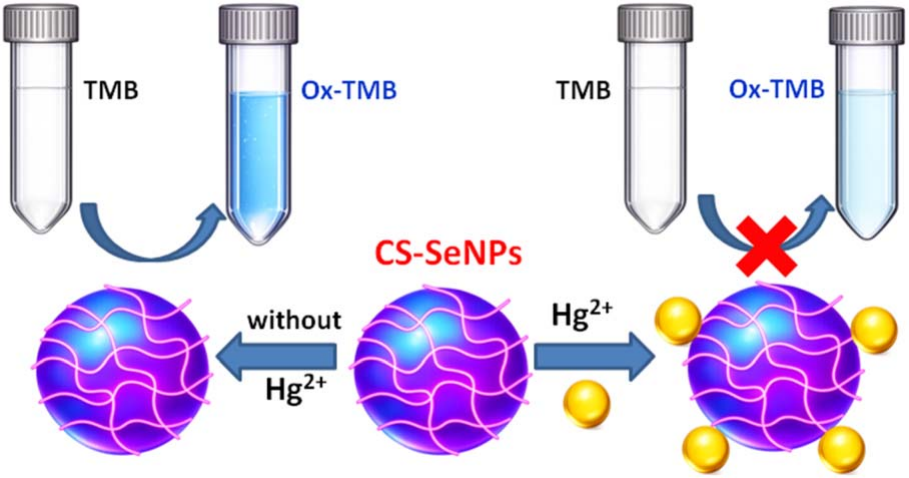
Schematic illustration of Hg^2+^ detection based on its inhibitory effect on the oxidase-like activity of CS-SeNPs.

SeNPs are appealing for biosensing in medical diagnostics and environmental monitoring, as well as for industrial analysis, because their semiconducting nature, redox activity, photoconductivity, and intrinsic hydrolysis are highly regulated by sensing interface properties.^90^ They also have a high surface area-to-volume ratio, which enables them to efficiently immobilise biomolecules and deactivate rapid signal transduction, thereby improving sensitivity and reliability in biosensing platforms. An important benefit of a-SeNPs is that they can be used as components of dual-mode and multifunctional sensing strategies. For example, a-SeNPs stabilized and surface-functionalized with carbon quantum dots (CQDs-SeNPs) were synthesised *via* the reduction of Na_2_SeO_3_ using ascorbic acid in the presence of β-cyclodextrin-modified N-doped CQDs, followed by dialysis purification. These CQDs-SeNPs functioned as a dual-mode sensor for Hg^2+^ detection, combining fluorescence quenching and mercury-triggered oxidase-like activity. Fluorescence quenching enabled sensitive Hg^2+^ detection in the range of 0.78–12.50 µmol L^−1^ with a detection limit of 0.14 µmol L^−1^, while at higher Hg^2+^ concentrations (25–50 µmol L^−1^), the formation of semimetallic HgSe activated catalytic oxidation of TMB, yielding a detection limit of 6.13 µmol L^−1^. This integrated sensing approach provided a broad detection window of 0.78–50 µmol L^−1^, a rapid response time of less than 20 minutes, and excellent performance in real water samples, highlighting the versatility of SeNP-based biosensors.^91^ The surface chemistry of SeNPs plays a decisive role in determining their catalytic and sensing performance. In another study, a-SeNPs stabilized with chitosan (CS), and sodium alginate, bovine serum albumin (BSA) were synthesised by reducing Na_2_SeO_3_ using ascorbic acid or GSH, producing spherical NPs with sizes ranging from 40 to 70 nm. Among these, CS-stabilized SeNPs exhibited significantly enhanced oxidase-like activity and enabled TMB oxidation without the need for H_2_O_2_ (Fig. 5), whereas BSA-stabilized SeNPs showed much weaker catalytic behaviour. Because of the significant affinity between selenium and mercury ions, Hg^2+^ selectively inhibited the catalytic reaction, enabling colourimetric detection in the range of 0.1–2.5 µM with a detection limit of approximately 0.12 µM.

Importantly, the sensor displayed excellent selectivity against a wide range of competing metal ions, including K^+^, Mg^2+^, Cu^2+^, Ca^2+^, Zn^2+^, Na^+^, Fe^3+^, Co^2+^, Pb^2+^, Cd^2+^, and Ni^2+^.^92^ Although the detection limit was not the lowest reported, CS-SeNPs offered notable practical advantages, including low cost, simplicity, and independence from unstable H_2_O_2_, making them competitive alternatives to noble-metal-based systems such as Pt, Pd, and Au.^93^

SeNPs are less well studied as glucose biosensing nanomaterials than noble-metal nanomaterials, but their potential is evident in current research. In a publication, glucose oxidase was adsorbed onto a carbon paste electrode coated with a SeNP-mesoporous silica (Se-MCM-41) composite. The crystallised enzyme retained good electrocatalytic activity and enabled efficient electronic transfer. The resulting glucose biosensor had a linear response over a concentration range of 0.01 to 2.0 mM, could operate over a wider pH range (4–8) and was more sensitive than conventional carbon paste electrodes. The sensor could also withstand a loss of about 91% of its original response over 10 days and show insignificant interference with other common substances, such as ascorbic acid and citric acid, suggesting a high level of stability and specificity.^94^ In broader applications, SeNPs have also been considered as biological signal modulators and probes beyond traditional chemical sensing applications. *Streptomyces* species biogenically synthesised SeNPs were demonstrated to inhibit quorum sensing in *Pseudomonas aeruginosa*, generating violacein inhibition zones of about 7.5 mm in depth and activity under sub-inhibitory concentrations (2.3592 µg mL^−1^) without impacting bacterial growth. These SeNPs outperformed biofilm formation (64–88%), pyocyanin production (65–90%), elastase activity (60–89%), and protease activity (83%) and down-regulated the expression of quorum-sensing-related genes, including up to 99.9%.^95^ Similarly, a-SeNPs synthesised *via* the reduction of Na_2_SeO_3_ using ascorbic acid and stabilized with hydroxyethyl cellulose were employed not as traditional chemical sensors but as tools to probe quorum sensing regulated biological behaviour. The a-SeNPs selectively disrupted bacterial communication without affecting cell viability. Violacein production dropped by about 80% in *Chromobacterium violaceum* ATCC 12472 at 250 mg Se per L, and biofilm formation in quorum-sensing-regulated biofilm was reduced by 60–70% in *Pseudomonas aeruginosa* at 100–250 mg Se per L, resulting in biofilm biovolume decline up to 98% as observed with confocal microscopy. These results justify the use of a-SeNPs to detect minute biological signalling events through optical and structural measures, thereby widening their operational scope beyond chemical detection to biological sensing and monitoring.^96^

### 5.6 Agriculture

a-SeNPs are also gaining popularity in the field of agriculture as a controlled delivery system of Se instead of a traditional fertilizer. Their relevance lies in supplying Se at very low concentrations where physiological benefits can be achieved without the toxicity often observed with selenite or selenate salts. The majority of agricultural research thus depends on seed priming, spraying foliage, or strictly controlled soil application rather than bulk soil incorporation. The primary benefit of SeNPs is that they provide a gradual release of selenium. They can affect plant metabolism at low doses without affecting normal growth. Their effects can be particularly noticeable under stressful conditions, during which the physiological stability of treated plants is preserved. In various experiments, the plants treated with a-SeNPs exhibit better antioxidant activity, including higher levels of superoxide dismutases and catalases, and lower levels of oxidative stress indicators. This biochemical adaptation helps maintain membrane integrity and enables growth under drought or heat stress.^97^

Soil-based evidence was clearly demonstrated by Gudkov *et al.*,^98^ who synthesised zero-valent a-SeNPs by pulsed laser ablation in aqueous medium and incorporated them into soil at 1–25 µg kg^−1^. Under optimal temperature, plant growth changes were minor. However, under heat stress (40 °C), treatment at 5–10 µg kg^−1^ significantly improved plant performance. Leaf surface area increased from 14 cm^2^ in stressed control plants to about 27–29 cm^2^. When the dose was increased to 25 µg kg^−1^, growth suppression occurred, confirming a narrow but effective concentration window. The internal behaviour of SeNPs was further elucidated by foliar application studies. In an *in vivo* investigation, Wang *et al.*^99^ examined how selenium derived from foliar-applied NPs is processed within plant tissues. Selenium was not detected as intact NPs in edible parts. Instead, it was transformed into selenium(iv) species and subsequently metabolised into organic forms, predominantly SeMet, which became incorporated into plant proteins. At appropriate application levels, biomass increased by approximately 10–40% compared with untreated controls, demonstrating efficient assimilation without NPs accumulation. This mechanism was further supported by field-scale findings in wheat. Field-scale results in wheat further supported this mechanism. Foliar application, *i.e.*, the direct spraying of SeNPs onto wheat leaves, at doses of 5, 10, and 20 g Se per ha significantly improved both grain yield and selenium biofortification. At 20 g ha^−1^, grain yield increased from 4283 to 5445 kg ha^−1^, corresponding to a 27% enhancement over control plants. In addition, grain selenium concentration, referring to the amount of selenium accumulated in the edible wheat grains, increased by 6–32 fold and reached approximately 0.89 mg kg^−1^ at the highest dose. Notably, 64–71% of the selenium accumulated in grains was present as SeMet, confirming efficient metabolic conversion into nutritionally relevant organic forms. The fertilizer use efficiency reached up to 59%, which was significantly higher than that of conventional selenate or selenite fertilizers. Importantly, intact a-SeNPs were not detected in harvested grains, indicating that selenium was mainly present in transformed ionic or organic forms rather than as particulate nanomaterials.^100^

### 5.7 Photocatalysis

**Fig. 6:**
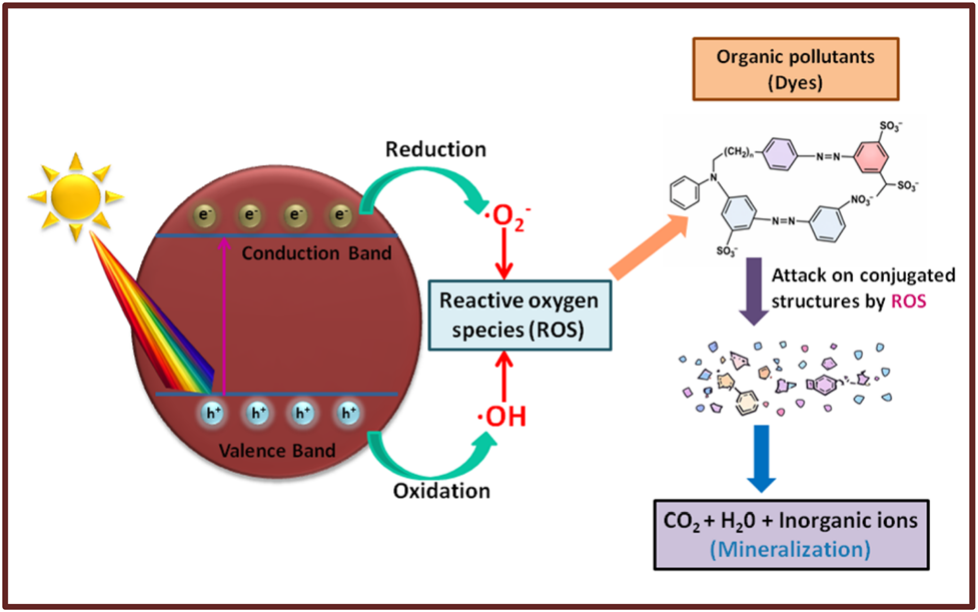
Schematic illustration of the photocatalytic degradation mechanism involving ROS generation and pollutant mineralization.

a-SeNPs have shown considerable potential as photocatalysts for the elimination of organic contaminants from aqueous systems. Their photocatalytic activity is mainly attributed to the semiconducting nature of selenium and its appropriate band gap energy. Selenium nanomaterials typically possess a band gap in the range of 1.8–2.0 eV, which supports visible-light absorption and photocatalytic activity. Generally, a-SeNPs exhibit a slightly higher band gap (∼2.0 eV) than c-SeNPs (∼1.8 eV) due to their disordered atomic arrangement and quantum confinement effects. Variations in band gap influence light absorption behaviour, electron–hole separation, and overall photocatalytic efficiency.^101^ This energy domain allows the SeNPs to make effective use of incident light and produce charge carriers that are used in redox reactions. When exposed to UV or visible light, electrons are promoted from the valence band to the conduction band, generating positively charged holes behind. The generated charge carriers subsequently take part in interfacial oxidation and reduction reactions: electrons reduce dissolved oxygen to produce superoxide reactive species (˙O_2_^−^) and holes oxidise water molecules or hydroxyl ions to generate highly reactive hydroxyl radicals (OH˙). The ROS produced in this reaction react with the conjugated structures of dye molecules, resulting in a slow process of decolourisation and disintegration into smaller, less damaging compounds, and in other cases, complete mineralization^102^ (Fig. 6).

Nazarzadeh *et al.*^103^ synthesised a-SeNPs using *Cuminum cyminum* seed extract and compared their photocatalytic activity with rhodamine B, methylene blue, and eriochrome black T under UV irradiation. They used 30 mg of the catalyst in a 10^−5^ M solution of dyes and monitored the gradual decrease in absorbance over time during irradiation. The gradual decrease in the intensity of the dye supported the effectiveness of photocatalytic degradation, which was explained by light-mediated charge separation and resulting ROS generation at the surface of SeNPs. Similarly, Sahoo *et al.*^104^ reported that a-SeNPs synthesised *via* biosynthesis efficiently degraded methylene blue under sunlight. The gradual decrease in absorbance at 660 nm indicated the decomposition of the dye. The paper also outlined that oxidative stress produced at the interface of SeNPs was directly correlated to the degradation process. The same ROS that led to the degradation of the dye also damaged the bacterial cell membrane, demonstrating the dual purpose of a-SeNPs as photocatalysts and antimicrobial agents.

In a separate report, Kamali *et al.*^105^ prepared a-SeNPs using *Nephelium lappaceum* seed extract and examined their photocatalytic properties under 365 nm UV light. Almost 69% degradation of methylene blue was attained in three hours. The increased performance was linked to efficient contact between dye molecules and the NP surface, which promoted charge transfer and increased ROS generation during irradiation.

### 5.8 Anti-inflammatory

Inflammation is a protective immune response that eliminates pathogens and repair tissues after injury. This chronic inflammatory response, however, is characterized by an overproduction of ROS and activation of inflammatory signaling pathways, leading to tissue damage and chronic inflammatory diseases.^106,107^ a-SeNPs have anti-inflammatory effects through ROS-scavenging, regulation of antioxidant enzyme (GSH-Px, SOD, CAT), NF-κB/NLRP3 signaling inhibition, reduction of inflammatory mediators (TNF-α, IL-1β, IL-6, NO, iNOS, COX-2, MPO), and modulation of macrophage/microglial activation, restoring immune homeostasis. In the ethanol-induced gastric ulcer model, *Bletilla striata* polysaccharide-functionalized a-SeNPs were found to be highly anti-inflammatory as evidenced by the reduction of gastric mucosal injury and restoration of SOD and GSH activity, and the reduction of the proinflammatory cytokines such as TNF-α, IL-1β, IL-6, NO, iNOS, MPO, ROS, and MDA.^108^ Likewise, the a-SeNPs derived from yeast showed strong immunomodulatory properties in cyclophosphamide (CPD)-induced immunosuppressed mice. SeNPs supplementation significantly improved GSH-Px (38.39%), SOD (13.52%) and TAC, and decreased MDA (31.52%) at the dose range 0.3–0.8 mg kg^−1^. Moreover, these NPs restored the liver, spleen, and kidney function and markedly improved the production of the immunoglobulin G (IgG), immunoglobulin M (IgM), and immunoglobulin A (IgA), suggesting restoration of immune homeostasis along with antioxidant protection.^109^ Moreover, surface-modified SeNPs exerted anti-inflammatory effects by inhibiting the NF-κB/NLRP3 inflammasome pathway, thereby suppressed the secretion of inflammatory cytokines, microglial activation, and changes in gut microbiota, demonstrating their anti-inflammatory effects in regulating immune cells during chronic neuroinflammation.^110^

## 6 Conclusions

a-SeNPs are increasingly being recognized as multifunctional nanomaterials that combine fundamental chemistry with advanced biomedical and technological applications. This random atomic arrangement of a-Se with increased surface area allows it to be more reactive, better interact with biological systems and have enhanced functionality compared to crystalline form. This is especially important in the area of antioxidant protection, antitumor, antimicrobial, bioimaging and biosensing where the structural characteristics are directly linked to the performance. Over the years, much progress has been achieved in the field of controlled synthesis methods, especially green and plant-mediated, which not only become less toxic, but also increase stability because of the application of natural stabilising agents. While these advances have been achieved, the practical use of a-SeNPs is still frequently linked with its metastable nature. The tendency to gradually progress towards more stable crystalline structures is a critical limitation, as it may reduce its bio-functionality. Therefore, it is not only a technological aspect, but also an important aspect to keep the amorphous state, by suitable stabilization strategies, to maintain their functional properties. On the other hand, reversible phase transition provides opportunities, where their properties can be switched by structural transformation under appropriate conditions, depending on the environment or physiology. a-SeNPs will arguably, in the future, move out from their conventional applications and move towards the field of adaptive and multifunctional nanomaterials. Their natural fluorescent properties and photophysical properties can be exploited in real-time biological imaging and tracking, sensitive detection and label-free monitoring of biological processes. At the same time, their responsiveness to external stimuli and flexibility of surface chemistry make them suitable for the design of smart therapeutic platforms, such as drug delivery, immunomodulation, and tissue regeneration. They are also being used for photocatalysis, energy harvesting and environmental remediation, which can make use of their defect structure. Another equally important aspect is to combine experimental studies with modelling. In particular, methods such as density functional theory (DFT), particularly in combination with machine learning methods, can offer insight into surface chemistry, defect chemistry and phase stability in amorphous materials and accelerate the design of high-performance materials.

## Author contributions

All authors contributed equally.

## Conflicts of interest

There are no conflicts to declare.

## Data Availability

This article is a review and does not report any new experimental data. All data discussed and analyzed are drawn from previously published studies, which are duly cited within the manuscript.
